# Real time detection of peptide–MHC dissociation reveals that improvement of primary MHC-binding residues can have a minimal, or no, effect on stability

**DOI:** 10.1016/j.molimm.2010.11.004

**Published:** 2011-01

**Authors:** Kim M. Miles, John J. Miles, Florian Madura, Andrew K. Sewell, David K. Cole

**Affiliations:** a Cardiff University, School of Medicine, Heath Park, Cardiff, UK; b Cellular Immunology Laboratory, Queensland Institute of Medical Research, Brisbane, Australia

**Keywords:** pMHC, peptide–major histocompatibility complex, MFI, mean fluorescence intensity, FACS, fluorescent activated cell sorting, SPR, surface plasmon resonance, TCR, T-cell receptor, Surface plasmon resonance (SPR), Peptide–major histocompatibility complex (pMHC), Heteroclitic anchor residue-modified peptide, pMHC stability, T-cells, Cancer vaccines

## Abstract

The majority of known major histocompatibility complex class I (MHCI)-associated tumor-derived peptide antigens do not contain an optimal motif for MHCI binding. As a result, anchor residue-modified ‘heteroclitic’ peptides have been widely used in therapeutic cancer vaccination trials in order to enhance immune responsiveness. In general, the improved stability of these heteroclitic complexes has been inferred from their improved immunogenicity but has not been formally assessed. Here, we investigated the binding of 4 HLA A*0201-restricted tumor-derived peptides and their commonly used heteroclitic variants. We utilized a cell surface binding assay and a novel robust method for testing the durability of soluble recombinant pMHCI in real time by surface plasmon resonance. Surprisingly, we show that heteroclitic peptides designed with optimal MHC binding motifs do not always form pMHCs that are substantially more stable than their wildtype progenitors. These findings, combined with our recent discovery that TCRs can distinguish between wildtype peptides and those altered at a primary buried MHC anchor residue, suggest that altered TCR binding may account for a large part of the increased immune response that can be generated by anchor residue-modified ligands. Our results further highlight the fact that heteroclitic peptide-based immune interventions require careful evaluation to ensure that wildtype antigen specificity is maintained *in vivo*.

## 1 Introduction

The T-cell receptor (TCR) governs T-cell mediated immunity through the recognition of short peptide fragments bound to major histocompatibility complexes (pMHCs) that are expressed on the surface of almost all nucleated cell types. Formation of stable pMHC class I (pMHCI) that can be transported to the cell surface for examination by the TCR depends on the binding affinity between the peptide and MHC. Peptides bind to MHC via interactions between individual residues and binding pockets in a groove on the MHC surface (Bjorkman et al., 1987; Guo et al., 1993). The most favorable residues at primary MHC anchor positions for the most common caucasian human leukocyte antigen (HLA), HLA A*0201, have been determined by elution and sequencing of self-peptides bound to HLA A*0201 at the cell surface (Falk et al., 1991). The current pool of HLA A*0201-restricted tumor epitopes that have been identified often contain sub-optimal primary HLA A*0201 anchor residues and are not thought to form optimally stable pMHCIs. As a result, a number of tumor specific ‘heteroclitic’ peptides, with modified optimal anchor residues, have been developed that theoretically improve the stability of tumor epitopes (Chen et al., 2000, 2005; Keogh et al., 2001; Parker et al., 1994; Parkhurst et al., 1996; Reche et al., 2004; Rosenberg et al., 2004; Terasawa et al., 2002). These peptides have been designed by introducing ‘optimal’ MHC anchor-residues at peptide position 2 and the peptide C-terminus based on: (i) peptide–MHC binding algorithms (Keogh et al., 2001) and (ii) intelligent design (Chen et al., 2000; Parkhurst et al., 1996). However, in most cases, the improved stability of these modified pMHCIs has been indirectly inferred due to the heightened T-cell immunogenicity of these molecules. Thus, the improved peptide–MHC affinity of these heteroclitic complexes has not been directly assessed. Here we have developed a surface plasmon resonance (SPR) pMHC stability assay that detects changes in mass at the surface of a gold plated sensor chip. This technology enables the determination of pMHCI half-life by detecting protein density at the sensor chip surface in real time. We have used this assay and a more commonly used cell surface binding assay to compare the stability of four MHCIs in complex with naturally expressed tumor epitopes in parallel with their anchor residue-modified heteroclitic variants. We show that: (i) pMHCI stability data from our biophysical assay are consistent with data independently generated from a cell-based assay that utilizes transport associated with antigen processing (TAP) deficient T2 cells (Henderson et al., 1992), (ii) altering a peptide in an attempt to improve MHC binding does not always correlate with an increase in stability over the wildtype progenitors, and (iii) commonly used peptide–MHC binding algorithms cannot always predict pMHC stability based on anchor residue-modifications.

## 2 Materials and methods

### 2.1 Generation of expression plasmids

The HLA A*0201 α chain, tagged with a biotinylation sequence, and β2m were inserted into separate pGMT7 expression plasmids under the control of the T7 promoter as previously reported (Cole et al., 2009) and the sequences were confirmed by automated DNA sequencing (Lark Technologies, Essex, UK).

### 2.2 Peptides

Lyophilized peptides were purchased from ProImmune (Oxford, UK). The peptides used in this study comprised: EBNA-1_407–415_ (HPVGEADYF), influenza M1_58–66_ (GILGFVFTL), NY-ESO-1_157–165_ (SLLMWITQC, SLLMWITQ**L**) (Chen et al., 2000), gp100_280–288_ (YLEPGPVA, YLEPGPV**V**) (Parkhurst et al., 1996), HER-1/neu_369–377_ (KIFGSLAFL, K**L**FGSLAF**V**) (Keogh et al., 2001), PSA_178–187_ (VISNDVCAQV, V**L**SNDVCAQV) (Terasawa et al., 2002).

### 2.3 Cell line

The mutant LCL × T-lymphoblastoid hybrid cell line, 174 × CEM.T2 (referred to as T2 cells) is a mutant antigen-presentation cell line (Henderson et al., 1992). These cells express stable human leukocyte antigen (HLA) class I molecules on their surface in the presence of exogenous peptides. Cells were maintained in Gibco RPMI 1640 (Invitrogen, Carlsbad, CA, USA) and supplemented with 10% heat inactivated foetal calf serum (FCS) (Invitrogen), 2 mM l-glutamine (Invitrogen), penicillin 50 Units/ml (Invitrogen) and streptomycin 50 μg/ml (Invitrogen).

### 2.4 T2-binding assay

The T2-binding assay was performed as previously described (Bell et al., 2009). Briefly, T2 cells were washed in AIM-V media (Invitrogen), and concentrated to 10^6^ cells/ml. The peptides were prepared in AIM-V media and serially diluted providing concentrations of 200 μM, 100 μM, 20 μM and 2 μM. The cells were mixed 1:1 with each peptide dilution to give a final volume of 200 μL and final peptide concentrations of 100 μM, 50 μM, 10 μM and 1 μM. A HLA A*0201 binding peptide, GILGFVFTL, and a non-HLA A*0201-restricted peptide, HPVGEADYF (HLA-B*3501), were included as positive and negative controls respectively. The assay was subjected to 10 min incubation at 37 °C 5% CO_2_ before incubation at room temperature overnight. Cells were then incubated for 2 h at 37 °C and stained with mouse anti-human HLA A*0201:RPE (Serotec, Oxford, UK). The cells were washed twice with PBS and analyzed using a BD FACS Canto II (BD Biosciences Immunocytometry Systems, San Jose, CA, USA), collecting 30,000 events in gate P1. Average MFI of the HLA A*0201:RPE antibody staining was used to measure the strength of binding in the T2 cell peptide–MHCI based assay. The MFI reading from the FACS Diva software was analyzed in GraphPadPrism using a log (agonist) vs. response non-linear curve to generate the logEC50 results.

### 2.5 pMHCI refolding and biotinylation

pMHCI was refolded and biotinylated as previously reported (Cole et al., 2007).

### 2.6 Real time SPR pMHC stability assay

The binding analysis was performed using a BIAcore 3000™ (GE Healthcare, UK) equipped with a CM5 sensor chip as previously reported (Cole et al., 2008). For SPR-based pMHCI stability assays, ∼500 RUs of biotinylated pMHCI were immobilized to streptavidin, which was chemically linked to the chip surface. A flow rate of 10 μl/min was established for 15,000 s. The biophysical results were analyzed by using Langmuir dissociation analysis which was then converted to half-life in seconds. The biophysical binding data from the BIAcore 3000™ was analyzed in the BIAevaluation software™ (GE Healthcare).

## 3 Results

### 3.1 Analysis of pMHC stability using TAP deficient T2 cells

**Fig. 1:**
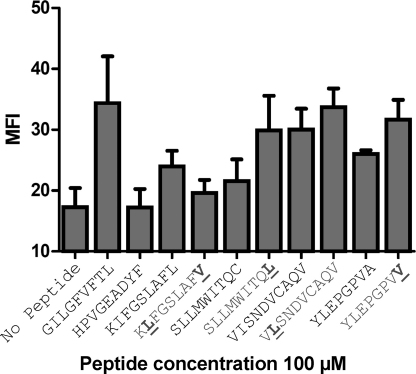
FACS analysis of pMHCI binding using TAP deficient T2 cells. We used TAP deficient T2 cells, which lack the ability to transport peptide fragments to the endoplasmic reticulum to form stable pMHCI, to analyze the stability of the natural and heteroclitic pMHCIs using FACS analysis. No peptide, and the HLA-B*3501-restricted EBNA-1_407–415_ (HPVGEADYF) peptide were used as negative controls of binding, and the HLA A*0201-restricted influenza M1_58–66_ (GILGFVFTL) peptide was used as a positive control of binding. The mean fluorescence intensity (MFI) of cell surface pMHCI staining with an anti-human HLA A2:RPE antibody revealed that the natural tumor antigen, KIFGSLAFL, was more stable than the heteroclitic variant, K**L**FGSLAF**V**, whereas the natural tumor peptides SLLMWITQC, YLEPGPVA and VISNDVCAQV were less stable than their heteroclitic counterparts: SLLMWITQ**L**, YLEPGPV**V** and V**L**SNDVCAQV. Data at a peptide concentration of 100 μM are shown. The binding assay was performed at a range of peptide concentrations (100 μM, 50 μM, 10 μM and 1 μM). LogEC50s (M) for this range are shown in Table 1. The mean standard deviation, representing 4 separate experiments, is shown as error bars.

The binding of 4 different HLA A*0201-restricted tumor-associated peptides was compared to their commonly used heteroclitic variants using a cell surface pMHCI stabilization assay. This assay made use of T2 cells that are deficient in the transporter associated with antigen processing (TAP) and that therefore lack the ability to transport peptide fragments into the endoplasmic reticulum to form stable peptide–HLA A*0201 complexes (Henderson et al., 1992). As a result, peptide–HLA A*0201 molecules expressed on the cell surface of T2 cells are generally of low stability. Incubation of T2 cells with exogenous peptide that binds to HLA A*0201 stabilizes the molecule so that surface pMHC expression levels can be quantitatively assessed (Bell et al., 2009). No peptide and the HLA B*3501-restricted EBNA-1_407–415_ (HPVGEADYF) peptide were used as negative controls for binding, and the HLA A*0201 restricted influenza M1_58–66_ (GILGFVFTL) peptide was used as a positive control for binding. Flow cytometric based analysis, using anti-human HLA A*0201:RPE antibody, revealed that the wildtype antigen, HLA A*0201-KIFGSLAFL, (logEC50 of −3.885 M), was more stable than the heteroclitic variant HLA A*0201-K**L**FGSLAF**V** (logEC50 of −3.389 M) that was designed to improve MHC binding (Fig. 1, Table 1). However, the wildtype pMHCIs, HLA A*0201-SLLMWITQC, HLA A*0201-YLEPGPVA and HLA A*0201-VISNDVCAQV were less stable than their heteroclitic counterparts (Fig. 1, Table 1), albeit by a relatively small degree. These data show that: (i) some heteroclitic peptides do not form more stable pMHCIs than their wildtype progenitors; and (ii) the difference in the stability between some heteroclitic vs. natural pMHCIs, particularly VISNDVCAQV and V**L**SNDVCAQV, could be very small. These unexpected findings underscored the need to develop a different technique to verify the observations of the T2-binding assay.

### 3.2 Real time SPR pMHC stability assay

**Fig. 2:**
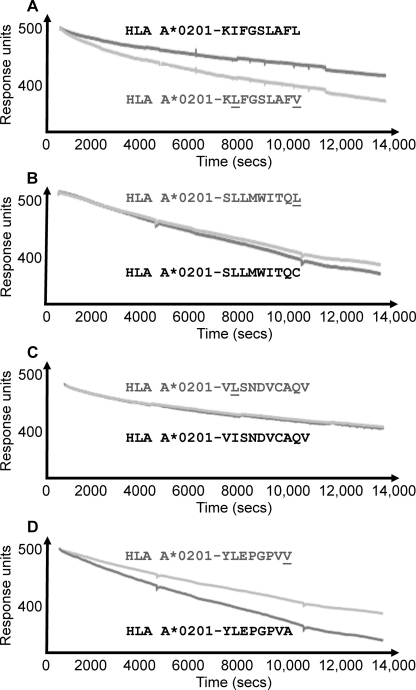
Real time SPR pMHC stability assay. We immobilized each wildtype and heteroclitic pMHCI pair to the surface of a CM5 chip using streptavidin-biotin coupling to assess the half-life of each complex in real time. In a similar trend to the T2-binding assay, we observed that; (A) the natural tumor antigen, HLA A*0201-KIFGSLAFL, had a longer half-life compared to the heteroclitic variant, (A) HLA A*0201-K**L**FGSLAF**V**, whereas the natural tumor antigens (B) HLA A*0201-SLLMWITQC, (C) HLA A*0201-VISNDVCAQV and (D) HLA A*0201-YLEPGPVA had faster off-rates compared to their heteroclitic counterparts, (B) HLA A*0201-SLLMWITQ**L**, (C) HLA A*0201-V**L**SNDVCAQV and (D) HLA A*0201-YLEPGPV**V**. Data are representative of 3 separate experiments.

One limitation of the T2-binding assay is its ability to reliably measure ligands with weak-to-medium affinities. To overcome this, we developed a highly sensitive biophysical assay to re-investigate the pMHCI stability of the wildtype and heteroclitic peptide cohort. The association of a peptide within the MHCI binding groove is required in order for the formation of a stable pMHCI molecule. Peptides that bind weakly to MHCI, usually because they do not contain optimal anchor residues, can result in unstable, short lived complexes. Thus, by investigating different pMHCIs using SPR, we were able to directly measure and compare the effect of peptide–MHCI binding on changes in mass at the surface of a CM5 sensor chip. In these experiments, we immobilized each wildtype and heteroclitic pMHCI pair to the surface of a CM5 chip using streptavidin–biotin coupling and measured the dissociation rate of each pMHCI in real time. A reduction in response units (RUs) (corresponding to a reduction in mass) over time was observed (Fig. 2). However, the loss of peptide from pMHCI complexes could not wholly account for this loss of mass due to the relatively small size of the peptide (∼1 kDa). Instead, we reasoned that because the MHCI heavy chain (α1, α2 and α3 domains) was chemically linked to the chip surface via a biotin–streptavidin interaction, the majority of this reduction was probably due to the dissociation of β2 microglobulin from the MHCI heavy chain. This effect was probably caused by destabilization of the pMHCI complex upon loss of the antigenic peptide. Thus, this decay process represents the stability of each pMHCI and infers the binding half-life of each peptide. We observed the same general pattern of pMHCI stability using SPR that was observed during the T2-binding assay (Fig. 1, Table 1). Importantly, we were also able to determine the half-life for each pMHCI in real time using our SPR pMHCI stability assay. The wildtype antigen, HLA A*0201-KIFGSLAFL had a longer half-life by 1.57 times compared to the heteroclitic HLA A*0201-K**L**FGSLAF**V** variant (Fig. 2A, Table 1) even though the latter is predicted to bind more strongly (Parker et al., 1994; Rammensee et al., 1999; Reche et al., 2004) (Table 2). Conversely, the wildtype HLA A*0201-SLLMWITQC, HLA A*0201-YLEPGPVA and HLA A*0201-VISNDVCAQV had faster off-rates compared to their heteroclitic counterparts, HLA A*0201-SLLMWITQ**L** (1.13 times slower), HLA A*0201-YLEPGPV**V** (1.47 times slower) and HLA A*0201-V**L**SNDVCAQV (1.10 times slower) (Fig. 2B–D, Table 1), confirming that, in these instances, modification of the peptide anchor residues improved peptide–MHC affinity. However, most noticeably in the cases of HLA A*0201-VISNDVCAQV and HLA A*0201-SLLMWITQC, the difference in stability between the wildtype peptide and the anchor improved peptide was minimal.

### 3.3 Comparison of peptide–MHC binding algorithms vs. experimental pMHC stability assays

A number of pMHC algorithms have been developed in order to predict: (i) peptide epitopes for different MHCs and (ii) peptide–MHC binding, and/or stability (Parker et al., 1994; Rammensee et al., 1999; Reche et al., 2004). We used these algorithms to determine the peptide binding score for the wildtype and anchor residue-modified heteroclitic peptide cohort that we had experimentally tested (Table 2). In agreement with the experimental data, the peptide binding scores calculated using the RANKPEP and SYFPEITHI peptide–MHC binding algorithms for wildtype HLA A*0201-SLLMWITQC, HLA A*0201-YLEPGPVA and HLA A*0201-VISNDVCAQV were only slightly lower than for their anchor residue-modified counterparts. The peptide binding scores calculated using the HLA_BIND algorithm also followed the same trend as the experimental data, but predicted a more substantial difference in pMHC binding for the wildtype HLA A*0201-SLLMWITQC, HLA A*0201-YLEPGPVA and HLA A*0201-VISNDVCAQV vs. the anchor residue-modified variants compared to RANKPEP and SYFPEITHI (Table 2). Interestingly, contrary to the experimental data, all of the peptide–MHC binding algorithms predicted scores for the wildtype HLA A*0201-KIFGSLAFL that were lower than for the heteroclitic HLA A*0201-K**L**FGSLAF**V** variant. This comparison underscores the importance of direct experimental determination of the effect of anchor residue-modifications on pMHC stability.

## 4 Discussion

A number of heteroclitic anchor residue-modified peptides have been designed in order to improve the interaction between the antigenic peptide and MHCI. These heteroclitic peptides have been shown to generate enhanced immunogenicity compared to their wildtype progenitors (Chen et al., 2000, 2005; Keogh et al., 2001; Parkhurst et al., 1996; Rosenberg et al., 2004; Terasawa et al., 2002). However, the proposed enhancement in stability of these pMHCs has not been formally tested. This is partly because there are currently no standardized methods to robustly compare the binding half-life of low/medium affinity peptide–MHC interactions. Here, we have designed and tested a real time biophysical assay for investigating the degradation of soluble recombinant pMHCI in comparison with a more widely used cell-based assay. We found that data generated from both assays were largely in agreement, but the SPR technique appeared superior in detecting subtle differences in low/medium affinity ligand dynamics. Additionally, compared with strictly end-point analysis of the cell-based assay, the SPR technique could measure detailed off-rate kinetics in real-time. Importantly, across both assays, we observed that the difference in stability between three-out-of-four of the heteroclitic pMHCIs was enhanced compared to their wildtype progenitors, although to an extremely small degree in the case of HLA A*0201-SLLMWITQ**L** and HLA A*0201-V**L**SNDVCAQV (Figs. 1 and 2, Table 1). We also used a number of peptide–MHC binding algorithms (Parker et al., 1994; Rammensee et al., 1999; Reche et al., 2004) to compare the theoretical stability of the wildtype and heteroclitic pMHCIs to our experimental findings. Although the peptide–MHC binding algorithms were able to predict enhanced binding for some of the anchor residue-modified heteroclitic peptides, they were unable to predict that the wildtype HLA A*0201-KIFGSLAFL was more stable than the heteroclitic HLA A*0201-K**L**FGSLAF**V** variant. The increase in binding in each case was relatively minor and it is difficult to reconcile how a <1.6-fold difference in pMHCI stability can result in an enhancement in immunogenicity of 10–100-fold (Keogh et al., 2001; Lienard et al., 2004; Parkhurst et al., 1996; Rosenberg et al., 2004, 1998; Speiser et al., 2002; Terasawa et al., 2002; Valmori et al., 1998; Yu et al., 2004). Our recent examination of TCRs specific for HLA A*0201-restricted peptides from Melan-A and preproinsulin demonstrated that TCRs can distinguish between wildtype peptides and those that have been altered at a ‘buried’ MHC anchor residue (Cole et al., 2010). These substantial differences in TCR/pMHC binding were confirmed by SPR and verified at the T-cell surface using pMHC tetramers (Cole et al., 2010). Here, we demonstrate that commonly used heteroclitic variant peptides designed to exhibit improved MHC binding do not necessarily substantially improve binding (Figs. 1 and 2). Nevertheless, it is well established that these peptides are substantially more immunogenic and prime significantly larger T-cell responses *in vitro* and *in vivo*. Unfortunately, the widespread use of heteroclitic peptides has been of limited clinical success (Rosenberg et al., 2004). In light of these data and our new findings, it is likely that the theoretical improved stability of heteroclitic pMHCIs (Table 2) is not the dominant underlying mechanism that governs the enhanced immunogenicity of these peptides. Since natural tumor epitopes are usually generated from self-molecules, highly reactive tumor specific T-cells for these structures are likely to be deleted in the thymus. However, because heteroclitic variants are slightly different to wildtype sequences, they may appear structurally ‘non-self’ to the immune system and appear as immunogenic structures. Indeed, it is clear that small changes in a peptide sequence can have very large effects on 3D structure, repertoire formation and immunogenicity (Turner et al., 2005; Valkenburg et al., 2010; Wieckowski et al., 2009). Likewise, it is also clear that changes in the amino acid side chain in an MHC-bound peptide can be transmitted along the peptide backbone and result in subtle structural differences at distal peptide residues (Reid et al., 1996; Theodossis et al., 2010). Thus, modification of peptide anchor residues could have an unpredictable knock-on conformational effect on the regions of the peptide that contact the TCR. Our findings suggest that, the design of some anchor residue-modified heteroclitic peptides may require careful revaluation in order to ensure that the T-cells generated by the altered antigen exhibit effective recognition of the natural tumor target.

In summary, we used a novel biophysical assay and a cell surface binding assay to formally test the binding of MHCI anchor modified heteroclitic peptides that have been used in the clinic. Surprisingly, we found that these pMHCIs were only moderately more stable than their wildtype counterparts; indeed in one case the modified pMHCI was less stable. The continued use of anchor modified peptides in the clinic is driven by their heightened immunogenicity. Our results suggest that the increased responses to these peptides cannot be wholly accounted for by their enhanced MHC binding. In light of our recent study that documents that TCRs can distinguish between peptides that use a different buried anchor residue (Cole et al., 2010), we suggest that the increased immunogenicity of many anchor modified peptides is most likely the result of these peptides creating non-self-pMHCI structures. These findings therefore have important implications for the use of altered peptide ligands (APL) in vaccination strategies and suggest that any APL intended for use in vaccination should be tested by *in vitro* priming to ensure that responding T-cells cross-react with the intended natural target.

## Figures and Tables

**Table 1:** Stability of wild-type, versus anchor residue-modified, peptide-MHCIs.

Peptide	^1^LogEC50 (M)	^2^*K*_off_ (s^−1^)	^3^Half-life (h)
KIFGSLAFL	−3.885	9.5 × 10^6^	20.1
K**L**FGSLAF**V**	−3.389	1.5 × 10^5^	12.8
SLLMWITQC	−3.532	2.2 × 10^5^	8.9
SLLMWITQ**L**	−4.365	1.9 × 10^5^	10.1
VISNDVCAQV	−4.301	1.1 × 10^5^	16.9
V**L**SNDVCAQV	−4.622	1.07 × 10^5^	17.8
YLEPGPVA	−3.587	3.3 × 10^5^	5.8
YLEPGPV**V**	−4.588	2.3 × 10^5^	8.5

^1^LogEC50 (M) for the T2-binding assays were generated using multiple concentrations (100 μM, 50 μM, 10 μM and 1 μM) for each peptide to calculate the half maximal response for each peptide–MHC interaction. The SPR biophysical stability data were analyzed by using Langmuir dissociation analysis^2^ which was then converted to half-life in hours^3^. Heteroclitic modifications are shown in bold and underlined.

**Table 2:** Predicted stability of wild-type and anchor residue-modified pMHCIs using pMHC binding algorithms.

Peptide	aRANKPEP binding score	bSYFPEITHI binding score	cHLA_BIND binding score
KIFGSLAFL	89	26	235
K**L**FGSLAF**V**	99	30	11394
SLLMWITQC	62	18	42
SLLMWITQ**L**	89	28	182
VISNDVCAQV	13	21	16
V**L**SNDVCAQV	16	23	118
YLEPGPVA	−3.7	n/a	n/a
YLEPGPV**V**	1.5	n/a	n/a

Heteroclitic modifications are shown in bold and underlined.

a Prediction of peptide binding score to HLA A*0201 using RANKPEP (Reche et al., 2004).

b Prediction of peptide binding score to HLA A*0201 using SYFPEITHI (Rammensee et al., 1999).

c Prediction of peptide binding score to HLA A*0201 using HLA_BIND (Parker et al., 1994).
